# Dog owner flea/tick medication purchases in the USA

**DOI:** 10.1186/s13071-018-3142-8

**Published:** 2018-11-06

**Authors:** Robert Lavan, Rob Armstrong, Kaan Tunceli, Dorothy Normile

**Affiliations:** 1 Outcomes Research, Animal Health, Center for Observational and Real-World Evidence, Merck & Co., Inc., Kenilworth, NJ USA; 2 Merck Animal Health, 2 Giralda Farms, Madison, NJ 07940 USA

**Keywords:** Ectoparasiticide, Dog, Veterinary practice, Purchases, Adherence, Duration, Flea, Tick

## Abstract

**Background:**

Veterinary clinic transaction records from the USA were examined to determine dog owner purchase patterns for three prescription ectoparasiticides. In-clinic purchases of formulations of fluralaner (with 12-week duration per dose) were compared with dog owner purchases of afoxolaner and spinosad (both with 4 week duration per dose) in a population of 231,565 dogs over a 12 month period. Prior studies in human and animal medicine have suggested that patients more closely adhere to prescriber dosing recommendations when they receive a longer-duration medication.

**Results:**

Veterinary clinic transaction records were examined for the period June 2014 through March 2017 using records from approximately 650 veterinary clinics. Ectoparasiticide purchase patterns were compared for two products (afoxalaner and spinosad) with monthly dosing and one product (fluralaner) with an extended (12 week) dosing interval. The average dog owner who obtained fluralaner purchased significantly more months of flea/tick protection (5.7 months) over the 12-month study period than the average dog owner that selected either afoxolaner (4.6 months) or spinosad (3.3 months). The proportion of dog owners who obtained only one dose of ectoparasiticide per 12-month period was 42% for fluralaner, 30% for afoxolaner and 37% for spinosad. The proportion of dog owners who obtained 2 doses or less per 12-month period was 67% for fluralaner, 52% for afoxoalaner and 67% for spinosad. Owners that obtained fluralaner were significantly more likely to obtain 7.0–12.0 months of flea and tick protection and significantly less likely to purchase 1.0–6.9 months compared with dog owners who purchased afoxolaner or spinosad.

**Conclusions:**

Dog owners who obtained a flea and tick medication with a longer duration of action acquired significantly more months of protection in a year than dog owners who obtained shorter duration (1 month) products. Dog owners were better able to adhere to veterinary recommendations on ectoparasites control with a longer-acting flea/tick medication.

## Background

Fleas and/or ticks are common ectoparasites in the USA and readily feed on companion animals. Owners of companion animals become aware of their pet’s parasitism when they see these pests in their home, on themselves or their families or on their pet. The clinical signs of ectoparasitism may appear as itching, rashes, lesions or infections.

Registered commercial products are available to effectively treat fleas and/or ticks on dogs and cats. Some products are available by prescription only while others can be obtained over the counter. Most products are dosed monthly and may be given as an oral, topical or injectable administration. Spinosad (Comfortis®, Elanco, Greenfield, IN, USA) is an oral prescription flea medication for dogs [1]. In 2014, a new isoxazoline class of parasiticide molecule was introduced to companion animal medicine in the USA, demonstrating a very high level of efficacy against fleas and ticks on dogs following systemic administration. Fluralaner (Bravecto®, Merck Animal Health, Madison, NJ, USA) and afoxolaner (Nexgard®, Merial, Duluth, GA, USA), are chews for dogs with different dosing intervals, for up to 12 weeks for fluralaner [2] and monthly for afoxolaner [3]. A topically administered fluralaner solution for dogs and cats (Bravecto Topical Solution, Merck Animal Health, Madison, NJ, USA) is also registered and commercially available.

A study in the USA investigated the flea and tick control recommendations of veterinarians and the experiences of pet owners who purchased fluralaner for flea and tick control on their dogs [4]. The participating veterinarians overwhelmingly recommended 12 months of flea and tick coverage for dogs; however, pet owner fluralaner purchases analyzed from over 9000 sales transactions for more than 5000 dogs found that the average pet owner obtained sufficient doses to cover the pet for 6.1 months [4]. USA studies prior to the commercial availability of fluralaner found that pet owners reported giving 4.0–4.5 doses per year of flea/tick medication, mostly provided as a monthly dose [5, 6]. The conclusion was that a longer duration treatment option was a significant improvement in coverage, although owners still fell short of veterinary recommendations.

Medications are only effective if they are taken as prescribed and missed doses can result in perceived and real treatment failures. Longer-acting medications require that fewer doses are administered to achieve adherence with the veterinary treatment recommendation. Previous research provides evidence that longer-acting medications improve human patient adherence to health provider recommendations [7–10]. There is an inverse relationship between dosing frequency and medication adherence in people, and significantly higher adherence rates are reported for medications with a longer duration of action and decreased dosing frequency [7–10]. This inverse relationship has been demonstrated across an array of drug classes including antibiotics, steroids and medications that treat respiratory disease, diabetes mellitus and hypercholesterolemia [11–14] and this relationship is partially responsible for the current trend toward longer-acting formulations in human medicine [13, 14]. A study involving veterinarians and dog owners in the USA suggests that a similar relationship exists between a requirement for less frequent dosing and increased adherence to veterinary flea/tick medication recommendations [4]. Findings from this study signal that longer retreatment intervals might contribute to improved dog owner adherence with veterinary flea/tick recommendations [4].

The objective of this study was to compare the duration (months per year) of flea and tick protection obtained by dog owners when they use either a longer-acting or a monthly treatment option. This study compared medication purchases for long-acting fluralaner (Bravecto®) and two monthly duration ectoparasiticides, afoxalaner (Nexgard®) and spinosad (Comfortis®). This comparison was undertaken over a much larger population of dogs than the previous investigation [4].

## Methods

This is a retrospective, observational study of veterinary transactional records from animal hospitals in the USA comparing dog owner obtained doses of three prescription oral products: fluralaner, afoxolaner and spinosad. Owner-obtained doses of these prescription products were used to estimate medication adherence for the products.

The raw data were downloaded from veterinary hospital transaction records through a proprietary medical records data collection service (VetInformatics, Inc., Rolling Meadows, Ill, USA). Collected data did not include proper names or addresses for dog owners, dog names or the identity of veterinary clinics. Code numbers were assigned to remove owner and patient identity while permitting match of serial transactions to individual dogs throughout the study period. The data vendor indicated that approximately 80% of the data came from the Southern and Midwest USA and the remainder (about 40,000 dogs) came from the Northeast and West USA as well as the USA protectorates of Puerto Rico and the Virgin Islands.

The study period started in July, 2014, and continued through March 31, 2017 and all three products were available throughout this period. Transactions for three oral prescription products (fluralaner, afoxalaner and spinosad) were compiled (Table 1). Non-prescription products were not included because they are available at many non-veterinary locations and it would not be possible to accurately represent pet owner purchases of these products using this database. Prescription products that included heartworm preventative efficacy were not included because the analysis focused only on ectoparasite control.

**Table 1:** Demographics of the study dogs

	Bravecto®	Nexgard®	Comfortis®
Number of dogs	58,731	118,594	54,240
Dog age (mean years ± SD)	5.3 ± 3.9	5.4 ± 3.8	5.8 ± 3.9
Dog age block (number of dogs, %)			
6 months to 1.0 year	11,940 (20.3)	22,427 (18.9)	8128 (15.0)
1.1–8.0 years	33,888 (57.7)	70,008 (59.0)	32,881 (60.6)
8.1–12.0 years	10,269 (17.5)	20,774 (17.5)	10,159 (18.7)
Over 12.1 years	2634 (4.5)	5385 (4.5)	3072 (5.7)
Dog body weight			
Mean weight ± SD (kg)	17.4 ± 11.7	18.1 ± 13.0	15.9 ± 12.7
Dog weight range (number of dogs, %)			
0–5.4 kg	10,360 (17.6)	22,889 (19.3)	14,100 (26.0)
5.5–11.3 kg	14,870 (25.3)	29,041 (24.5)	14,136 (26.1)
11.4–22.6 kg	11,740 (20.0)	20,780 (17.5)	8092 (14.9)
22.7–45.3 kg	20,755 (35.3)	42,401 (35.8)	15,986 (29.5)
45.4–90.7 kg	448 (0.8)	2869 (2.4)	1171 (2.2)
90.8+ kg	16 (0.0)	27 (0.0)	7 (0.0)
Missing data	542 (0.9)	587 (0.5)	748 (1.4)

Inclusion criteria applied to the data were: dog owners identified as “pure users” of a single flea or flea and tick product based on at least one full year history of using a single ectoparasiticide medication; dogs older than 6 months. Exclusion criteria applied removed non-canine species; and duplicate entries. Additional criteria applied were to ensure that dose counts were correct, especially with multi-dose packs, and to ensure that patient records represented transactions for one dog and not multiple dogs. Transactions for multiple dogs were excluded by eliminating records where the owner obtained more than 12 months of flea/tick coverage for an individual dog in a single transaction or if the owner obtained more than 24 months of coverage in a 12-month period. The exclusion was set at this apparently high level to allow for the situation in which an owner acquired the maximum of 12 months of protection for the one year and then obtained sufficient protection for the subsequent 12 months before the current 12-month period had ended.

To record and analyze transactions from individual owners and dogs, an Index Date (ID) was defined as the date of the first transaction for a specific dog owner in the database during the study period. The ID needed to be prior to March 31, 2016 to allow for a full 12-month window to acquire additional doses. The follow-up period for that specific owner was then defined as the 12 calendar months following the ID. This follow-up period looked at only transactions for the index drug (f the initial ectoparasiticide product obtained by the owner). These transactions were used to provide duration of coverage estimate by converting the doses obtained into a protection duration defined based on the registered product prescribing instructions in the USA [1–3]. Each fluralaner dose was calculated as providing 84 days (12 weeks × 7 days/week) of protection while each afoxolaner and spinosad dose was calculated to provide 30 days flea and tick protection duration. The potential total duration of coverage in the 12 months following the ID was then determined by adding up the total doses obtained in the follow-up period that could also be practically administered in the follow-up period. Therefore, a dose obtained late in the 12 month follow-up period only counted for a fraction of the last month, e.g. if the first monthly dose was purchased on January 1 and the last dose was purchased in December 15 of the same year, then the dose purchased in December would be considered to have provided 15/31 days of ectoparasite protection. Doses, or proportion of doses, that would have provided flea/tick protection after the end of the 12-month period following the ID were not included in the calculation of the duration of flea/tick protection. All owners were assumed to have administered all doses they obtained at the correct time and at the correct consecutive intervals if multiple doses were obtained in one recorded transaction.

Dog age, age block, body weight and weight range were compared between product groups with descriptive statistics, which included frequencies, percentages, means and standard deviations. The amount of yearly flea/tick coverage obtained by dog owners in a year was expressed as a population mean, standard deviation and percentage for each product group. Means were compared across groups using a Chi-square test with significance set at *P* < 0.05.

## Results

The unfiltered database included 515,102 dogs whose owners received one of the three ectoparasiticide products considered in this study between July 2014 and March 2017. The filtered database, after application of all inclusion and exclusion criteria, included 231,565 dogs and their transaction records for purchases of fluralaner, afoxalaner and spinosad.

**Fig. 1:**
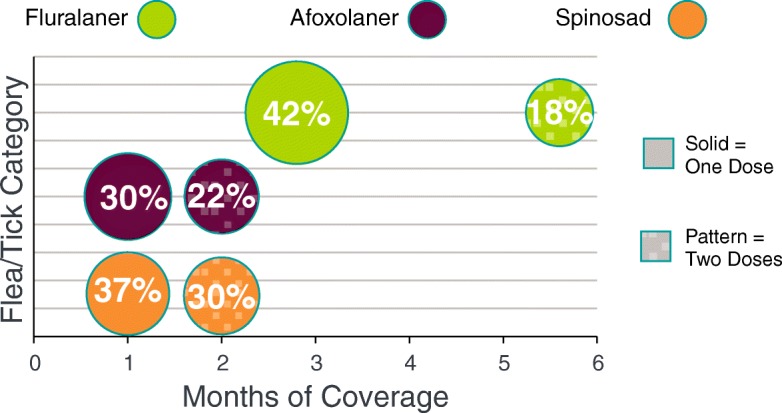
The proportion of USA dog owners receiving either one or two doses of ectoparasiticide products in a 12-month period

Demographic information for dogs in this study (Table 1) is broken out by ectoparasiticide product. These data include the number of dogs, the age and age block, the weight and weight range block. Because of the large sample size, the dog age and weight were significantly different between flea/tick products even though the differences were minimal thus clinically comparable. Across groups, the difference in average age was 0.5 year (about 10%) and in weight was 5 pounds (about 12%). Distribution by age block and weight range block were similar across ectoparasiticide product with the most frequently reported age block of 1–8 years and weight range block 50–100 pounds. Dog owners who obtained fluralaner obtained significantly more months of coverage in a year than owners who purchased either afoxolaner (*χ*^2^ = 5537.81, *df* = 1, *P* < 0.0001) or spinosad (*χ*^2^ = 19,593.55, *df* = 1, *P* < 0.0001) (Table 2). On average, owners obtained 5.7 usable months of flea and tick protection with fluralaner; 4.6 usable months of flea protection with afoxolaner or 3.3 usable months with spinosad over a 12 month period. This translates into a gain in the average duration of protection of 24% with fluralaner over afoxolaner and 73% for fluralaner over spinosad. Each fluralaner dose is labeled to provide flea/tick protection for 12 weeks, with 4.3 doses providing 12 months of coverage while for monthly products 12 doses provide 12 months of coverage. A large proportion of dog owners acquired only 1 dose of flea/tick medication per year (Table 3, Fig. 1) while a smaller proportion of owners purchased 2 doses in a year. Over the year period, 42% of pet owners who received fluralaner purchased one dose, providing 12 weeks (2.8 months) of flea/tick coverage. Approximately 22% of dog owners who purchased fluralaner purchased a second dose, providing a total of 24 weeks (or 5.6 months) of flea/tick coverage. For afoxolaner, approximately 30% of dog owners obtained 1 month of coverage and 22% obtained 2 months of coverage. For spinosad, approximately 37% obtained 1 month of coverage and 30% obtained 2 months of coverage for their dogs.

**Table 2:** Purchased ectoparasiticide doses by USA dog owners in a 12-month period

	Fluralaner	Afoxolaner	Spinosad
Number of dogs	58,731	118,594	54,240
Mean doses acquired (mean ± SD)	2.3 ± 1.4^a^	5.3 ± 5.0^b^	3.5 ± 3.5^c^
Months of possible protection (mean ± SD)	5.7 ± 3.1^a^	4.6 ± 3.7^b^	3.3 ± 2.9^c^

Means with different superscripts differ significantly at *P* < 0.05. Fluralaner *versus* afoxolaner (*χ*^2^ = 5537.81, *df* = 1, *P* < 0.0001) or spinosad (*χ*^2^ = 19,593.55, *df* = 1, *P* < 0.0001)

**Table 3:** USA dog owner yearly purchases of flea and tick protection

Months of coverage acquired	Fluralaner (%)	Afoxalaner (%)	Spinosad (%)
1.0–1.9	Not applicable	30.3	37.2
2.0–2.9	42.1^a^	21.6	30.1
3.0–3.9	1.6	1.1	1.2
4.0–4.9	1.5	4.5	6.3
5.0–5.9	22.1	15.6	13.0
6.0–6.9	2.1	2.4	1.7
7.0–7.9	2.2	3.9	2.2
8.0–8.9	10.3	2.7	1.5
9.0–9.9	2.4	3.7	1.3
10.0–10.9	3.4	1.9	0.7
11.0–11.9	8.4	6.6	3.0
12.0	4.0	5.6	1.7

^a^One dose of Bravecto provides 12 weeks of protection or 2.79 months

Pet owners that obtained fluralaner were significantly more likely to purchase 7.0–12.0 months of coverage and significantly less likely to purchase 1.0–6.9 months of coverage compared to pet owners that purchased either afoxalaner (*χ*^2^ = 756.04, *df* = 1, *P* < 0.0001) or spinosad (*χ*^2^ = 6935.64, *df* = 1, *P* < 0.0001) for their dogs (Table 4).

**Table 4:** Proportion of dog owners that purchased 1–6 months and 7–12 months of flea and tick medication in a year

Months of coverage acquired	Fluralaner (%)^a^	Afoxalaner (%)^b^	Spinosad (%)^c^
1.0–6.9	69.4	75.5	89.6
7.0–12.0	30.6	24.5	10.4

Different superscripts indicate statistically significant differences at *P* < 0.05 (Chi-square test). Fluralaner *versus* afoxalaner (*χ*^2^ = 756.04, *df* = 1, *P* < 0.0001) or spinosad (*χ*^2^ = 6935.64, *df* = 1, *P* < 0.0001)

## Discussion

This study found that dog owners obtain significantly more months of flea and tick protection during the year when they obtain a 12-week duration ectoparasiticide rather than a monthly duration ectoparasiticide. Dog owners that use the 12-week duration fluralaner obtained a 24% increase in months of protection compared with dog owners that obtained monthly afoxolaner and a 73% increase compared with dog owners that obtained monthly spinosad. This finding confirms the results of an earlier study of fluralaner use based on a smaller population of dogs in the USA [4] and a recent comparative study in Spain [15].

These studies also show that, although owners obtain more months of coverage with a longer duration of efficacy ectoparasiticide, most still fall short of the months of protection recommended by veterinarians [4]. In the present study, months of protection obtained by the dog owner fell short of average veterinarian recommendation by 53% for fluralaner, 62% for afoxalaner and 71% for spinosad. Approaches that veterinarians could adopt to increase dog owner adherence to their treatment recommendations involve reducing the barriers to acquiring and administering flea and tick treatments, educating dog owners about the health effects of flea and tick infestation and providing a treatment option that owners consider convenient [16]. Use of a flea and tick product with a longer duration of protection helps veterinarians increase dog owner adherence with veterinary recommendations.

The analysis in this investigation permitted a detailed breakdown of owner ectoparasiticide purchases over the 12 months following their initial prescription. Many dog owners purchased one dose of ectoparasiticide in this 12-month follow-up period (Table 3) including 42% of fluralaner users, 30% of afoxalaner users and 37% of spinosad users. There is a significant biological difference in the impact of 12 weeks of flea control compared with one month related to the nature of the flea life cycle within the household. One dose providing 12 weeks protection duration eliminated the flea population on dogs in simulated households [17–19] and in a field investigations [20]. However, a single dose of a product that provides a one-month duration of protection, such as afoxolaner or spinosad, cannot resolve an existing flea infestation [21]. Flea infestation treatment recommendations focus on providing protection for 90 days and require application of approximately three ectoparasiticide doses, and usually more, when the treatment interval is one month [22]. Administration of only one dose of a monthly product will certainly result in a rapid flea population rebound as the efficacy tapers and the dog becomes re-infested.

A smaller proportion of dog owners obtained two doses of a flea and tick product (Fig. 1). Owners obtaining two doses would get 5.6 months of flea and tick coverage with fluralaner or two months with afoxolaner or spinosad. The results do not evaluate the occurrence or duration of any gaps between administration of the two doses obtained by the owners and it is assumed for this analysis that all obtained doses were given at the correct time. Under this assumption, two doses of a monthly product provide a shorter duration of flea and tick protection than a single dose of a product with a 12-week duration. Additionally, if there is a delay between administrations of monthly doses then this could lead to further impacts on the apparent efficacy.

Ectoparasiticides for dogs are often packaged with 3, 6 and/or 12 doses in a single package to help the owner obtain sufficient doses to comply with veterinary parasite control recommendations. In spite of this, dog owners in this study who obtained afoxalaner or spinosad were significantly more likely to take home 1.0–6.9 months of coverage and less likely to take home 7.0–12.0 months of coverage, compared with owners who obtained fluralaner (Table 4). Therefore, dog owners prescribed fluralaner were significantly more likely to obtain sufficient months of protection to be able to adhere to veterinary recommendations for year-round flea and tick protection. A small fraction of dog owners acquired 12 months of flea and tick protection, enough to be fully adherent to veterinary recommendations (Table 3).

The most important limitation of transactional data for assessing dog owner adherence is that a dose obtained by the owner is not necessarily an administered dose. A history of purchased doses provides an estimate of the maximum adherence that a dog owner might achieve. The true adherence will be lower than the total potential months of coverage obtained by the owner. However, while all pet owners were vulnerable to delivering late or missed doses, the opportunity for missing a dose is reduced with a longer-acting medication because of the fewer doses required per unit time. Therefore, true adherence is likely to be closer to the potential months of coverage for a long duration treatment than for a monthly duration treatment.

## Conclusions

Dog owners who obtain an ectoparasiticide with a longer duration of protection will demonstrate improved adherence to flea and tick control recommendations and increased duration of ectoparasite coverage during the year.

## Abbreviations

CORE: The Merck Center for Observational and Real-World Evidence
ID: Index date (the first date that an ectoparasiticide was purchased in the 12-month purchase examination period)
OTC: Over the counter (available without a prescription)
